# Evaluation of the relationship of posterior tibial slope with gender and age in Turkish population with 3 different methods

**DOI:** 10.1186/s12891-024-07209-3

**Published:** 2024-01-30

**Authors:** Seyhmus Kavak, Sehmuz Kaya

**Affiliations:** 1 Department of Radiology, University of Health Sciences, Gazi Yasargil Training and Research Hospital, Elazig Road, 10th km Uçkuyular Location, Kayapınar, Diyarbakir, 21070 Turkey; 2 Dursun Odabaşı Medicine Center, Department of Orthopedics and Traumatology, University of Yüzüncü Yıl, Van, Turkey

**Keywords:** Posterior tibial slope, Turkish people, X-Ray

## Abstract

**Background:**

This study aimed to reveal the posterior tibial slope (PTS) angle with 3 different methods in a large case group in the Turkish population. In addition, the reproducibility of the measurement methods used was questioned while determining the age groups, gender and side relationship of this angle.

**Materials and methods:**

In our retrospective study, radiographs of both knees were evaluated in all 610 patients (344 women, 56.4%) aged 25–65 years. PTS angles were measured by a radiologist and an orthopedist using anterior tibial cortex (ATC), posterior tibial cortex (PTC) and proximal tibial anatomical axis (PTAA) methods. The relationship of these angles with age group and gender, and the intra-class and inter-class correlations of all three methods were evaluated.

**Results:**

The mean and standard deviation (SD) of PTS angle was 11.03 ± 2.33° with ATC method, 6.25 ± 2.22° with PTC and 8.68 ± 2.16° with PTAA, and the difference was significant (*p* < .001). In the evaluation according to age groups, the highest mean PTS angles were detected in cases aged 25–35 (9.63 ± 1.97° [mean ± SD] by PTAA method), and there was a significant difference in comparison with other age groups (*p* < .05). In comparison with age groups, higher mean PTS angles were found in women and on the right side, but the difference was not statistically significant (*p* > .05). The intraclass and interclass correlation coefficient (ICC) of all three methods was excellent (ICC > 0.91).

**Conclusion:**

This study emphasizes that the mean PTS angle in Turkish population is higher than the angle values ​​recommended by prosthesis manufacturers, and factors such as patient age and gender should be calculated in order to ensure more effective prostheses to be applied to patients.

## Background

The posterior tibial slope (PTS) is quantified as the angle between the vertical line representing the anatomical axis of the tibia and the tangent line representing the slope of the tibial plateau from the anterior to posterior [1–7]. The angular slope of the tibial plateau can have an impact on various aspects of knee function, such as range of motion, flexion gap, knee joint stability, anterior cruciate ligament (ACL) stability and posterior femoral rollback [8, 9]. Accurate measurement of the PTS is crucial for maximizing the benefits of surgical interventions including total knee arthroplasty (TKA), high tibial osteotomy, and ACL reconstruction [10, 11]. The relationship between ACL injuries and PTS angle has been reported in many studies [12–16]. An elevated tibial slope is associated with a higher likelihood of increased tension on the ACL, which can potentially lead to ACL tear. Conversely, a reduced tibial slope is anticipated to generate tension and potential damage to the posterior cruciate ligament, producing a similar effect observed with increased tibial slope and the ACL [13, 14, 17, 18]. With similar mechanisms of action, an increase in PTS may lead to abrasion of the polyethylene prosthesis placed with TKA and ultimately to aseptic loosening of the prosthesis. A reduced tibial slope can result in a forward shift of the line of force, reduced range of knee joint motion and flexion, and increased postoperative stiffness [2, 19–22].

PTS, being a critical reference point for achieving optimal mechanical and anatomical alignment, has been measured using various methods and imaging techniques [5, 6, 23–25]. Although X-ray imaging of the knee is the most commonly used modality, computed tomography (CT) and magnetic resonance imaging (MRI) are also utilized for PTS measurement. X-ray imaging techniques employ various measurement methods such as the anterior tibial cortex (ATC), posterior tibial cortex (PTC), proximal tibial anatomical axis (PTAA), central anatomical axis, and fibular shaft axis to assess the PTS [7, 10, 26]. Variations in reported PTS values can arise from differences in the measurement methods employed to assess PTS as well as the inclusion of differed patient populations across research studies. It is argued that factors such as ethnicity, gender and age can have a significant impact on the PTS value [10, 26–31].

The primary aim of this study was to determine the mean PTS angles in the Turkish population and to investigate potential associations between gender and age groups with the measured PTS angles in both knees. The second objective is to compare the mean PTS angles obtained using various techniques and assessing the repeatability of these methods.

## Materials and methods

### Patient selection

Between January 2020 and January 2023, patients between the ages of 25–65 years who applied to the emergency department and orthopedic outpatient clinic of our hospital and underwent lateral radiography of both knees were retrospectively evaluated from Picture Archiving and Communication System (PACS). From the selected patient population, individuals who exhibited intact bone structure, no prior or current tibial fractures, and no documented systemic conditions associated with bone degeneration were identified. A total of 610 patients and 1220 knee lateral radiographs were analyzed in this study. The inclusion criteria for radiographs included a difference of less than 5 mm between the posterior segments of the femoral condyles and clear visualization of the knee joint space, ensuring high-quality examinations. Patients were categorized into four age groups: group 1 (25–35 years), group 2 (36–45 years), group 3 (46–55 years), and group 4 (56–65 years).

### Image acquisition and radiologic evaluation

Images of all patients were obtained using GXR-SD (DRGEM Healthcare, Gyeonggi-do, South Korea) devices. Images of appropriate quality were evaluated using OsiriX MD software and PTS measurements were performed.

**Figs. 1:**
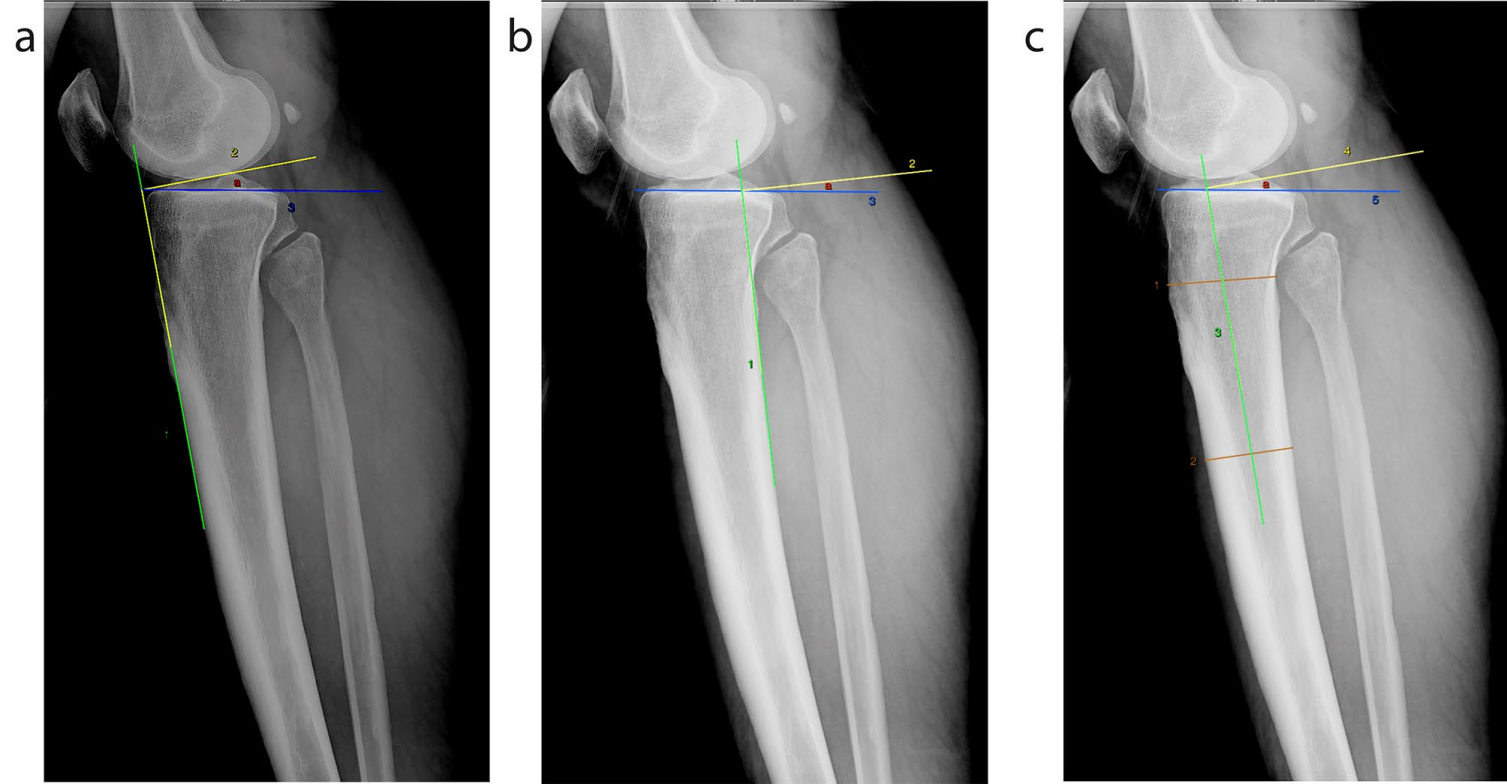
**a-c:** Posterior tibial slope measurement methods in left knee lateral radiograph. Anterior tibial cortex **(a)**, posterior tibial cortex **(b)**, and proximal tibial anatomical axis **(c)**

PTS angle was measured from both knees of all patients by using ATC, PTC, and proximal tibial anatomical PTAA methods. In the ATC method, the first line was drawn tangent to the anterior edge of the proximal tibia to represent the longitudinal axis of the tibia. The second line was then drawn at right angles to the first line. Finally, the third line was drawn tangent to the plateau, connecting the anterior and posterior endpoints of the tibial plateau. The angle between the second and third line was considered as PTS (Fig. 1a). In the PTC method, the first line was drawn tangent to the posterior edge of the proximal tibia to represent the longitudinal axis of the tibia. The second and third lines were drawn as in the ATC method and the angle between these lines was considered as PTS (Fig. 1b). Finally, in the PTAA method, two lines were drawn on the tibial corpus 5 and 15 cm from the tibial plateau to connect the ATC and PTC. A third line was drawn through the midpoint of both of these lines, representing the longitudinal axis of the tibia. Then a fourth line was drawn at right angles to this line. Finally, the angle between the fifth line drawn on the tibial plateau and the fourth line was considered as PTS (Fig. 1c). The measurements were performed independently by a radiologist and an orthopedist. The radiologist performed PTS measurements of both knees of all patients included in the study, while the orthopedist measured PTS values of 50 randomly selected patients. A blinded radiologist repeated PTS measurements for 50 randomly selected patients two months after the initial measurement date.

### Ethical approval

Approval was obtained from the ethics committee of Health Sciences University Gazi Yaşargil Training and Research Hospital​(Reference number and date: 414/05.26.2023). The retrospective study design waived the requirement to obtain informed consent from patients.

### Statistical analysis

SPSS version 23.0 was used to analyze the collected data. After testing for normal distribution using Kolmogorov–Smirnov and Shapiro–Wilk tests, descriptive statistics such as frequency analysis and percentage analysis were used for categorical variables, and mean and standard deviation were used for continuous variables. The mean PTS angles obtained using three different measurement methods were analyzed, and the differences between the mean values of the two knees were assessed using T-test. A two-way analysis of variance (ANOVA) test was employed to examine the association between PTS values, as measured by three different methods, and the variables of gender and age groups. A post hoc test was then conducted to determine the direction of significance. Intraobserver and interobserver correlations of PTS values measured by three different methods from the right knee were evaluated using intraclass correlation coefficient (ICCs). The interpretation of the degree of agreement for different ICC values is as follows: ICC ≤ 0.20, poor; 0.2 < ICC ≤ 0.4, poor–moderate; 0.4 < ICC ≤ 0.6, moderate; 0.6 < ICC ≤ 0.8, significant; and ICC ≥ 0.8, excellent [11]. A significance level of *p* < .05 was used as the threshold for statistical significance in all the employed statistical analyses.

## Results

**Figs. 2:**
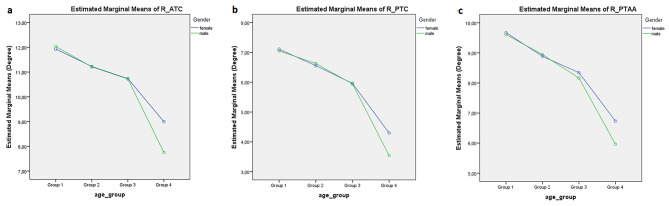
**a-c**: Gender and age group relationship of posterior tibial slope in the right knee according to three measurement methods. Anterior tibial cortex **(a)**, posterior tibial cortex **(b)** and proximal tibial anatomical axis **(c)** method

A total of 610 patients were included in the study and PTS angles were measured from two knees of each patient using ATC, PTC, and PTAA methods. Of the patients, 344 were women with a mean age of 45.02 ± 9.94 (mean ± SD) years and 266 were men with a mean age of 37.21 ± 10.45 years. The mean PTS angle of the right knee in women was 10.89 ± 2.41 with the ATC method, 6.14 ± 2.35 with PTC and 8.55 ± 2.27 with PTAA, and the mean PTS angle of the right knee in men was 11.33 ± 2.38 with the ATC method, 6.52 ± 2.28 with PTC and 8.95 ± 2.20 with PTAA. When analyzing all patients, the mean posterior tibial PTS angle measured with the PTAA method was 8.73 ± 2.25 (SD) in the right knee and 8.66 ± 2.08 (SD) in the left knee. Although the slope angle was slightly higher in the right knee, the difference was not statistically significant. *(p* = .12) (Table 1). When examining the patients based on age groups, the mean PTS angle measured using the PTAA method was 9.67 ± 1.91 (SD) for women in group 1 and 9.6 ± 2.01 (SD) for men in the same age group (Table 2). When the patients were evaluated by two-way ANOVA test based on age group and gender, mean PTS angles measured by PTAA method did not show a significant statistical relationship with gender *(p* = .257; F: 1.289)(Fig. 2a–c), while a significant relationship was found with age groups *(p* < .001; F: 35.014)(Table 3). According to age groups, higher PTS angles were found in younger patients (Table 4). PTS measurements of both observers provided the highest agreement with the PTAA method (ICC: 0.987; 95% CI (0.97–0.99)), whereas the agreement with the other two methods was excellent (Table 5).

**Table 1:** Posterior tibial slope measurement in the right and left knees with 3 different methods according to gender

				Measurement Method				
Gender	N	Age (Year) (Mean±SD)	R-ATC (Mean±SD)	R-PTC (Mean±SD)	R-PTAA (Mean±SD)	L-ATC (Mean±SD)	L-PTC (Mean±SD)	L-PTAA (Mean±SD)
Female	344	45.02 ± 9.94	10.89 ± 2.41	6.14 ± 2.15	8.55 ± 2.27	10.81 ± 2.2	6.01 ± 2.11	8.48 ± 2.01
Male	266	37.21 ± 10.45	11.33 ± 2.38	6.52 ± 2.28	8.95 ± 2.2	11.23 ± 2.31	6.43 ± 2.16	8.86 ± 2.15
Total	610	41.61 ± 10.87	11.08 ± 2.4	6.31 ± 2.33	8.73 ± 2.25	10.99 ± 2.26	6.19 ± 2.13	8.64 ± 2.08

*SD*: Standard deviation, *R-ATC*: Right knee anterior tibial cortex, *R-PTC*: Right knee posterior tibial cortex, *R-PTAA*: Right knee proximal tibial anatomic axis, *L-ATC*: Left knee anterior tibial cortex, *L-PTC*: Left knee posterior tibial cortex, *L-PTAA*: Left knee proximal tibial anatomic axis

**Table 2:** Mean PTS angles in the right knee by gender and age groups

Sex		Group	Mean*	Std. Deviation	N
Female		Group 1	9.672	1.913	70
		Group 2	8.885	1.837	82
		Group 3	8.341	2.293	152
		Group 4	6.732	2.370	40
		Total	8.554	2.275	344
Male		Group 1	9.608	2.007	138
		Group 2	8.943	1.673	60
		Group 3	8.163	2.171	52
		Group 4	5.962	2.596	16
		Total	8.956	2.209	266
Total		Group 1	9.630	1.972	208
		Group 2	8.909	1.763	142
		Group 3	8.296	2.258	204
		Group 4	6.512	2.438	56
		Total	8.730	2.253	610

*Group 1*: Between 25 to 35 years old, *Group 2*: Between 36 to 45 years old, *Group 3*: Between 46 to 55 years old, *Group 4*: Between 56 to 65 years old

*Posterior tibial slope measured by the proximal tibial anatomic axis method

**Table 3:** Analysis of the posterior tibial slope measured by three different methods with two-way ANOVA test

		R-ATC				R-PTC				R-PTAA			
Source	df	Type III Sum of Squares	Mean Square	**F**	***p***	Type III Sum of Squares	Mean Square	**F**	***p***	Type III Sum of Squares	Mean Square	**F**	***p***
Corrected Model	7	555.253^**a**^	79.322	16.064	0.000	442.334^**b**^	63.191	13.236	0.000	495.273^**c**^	70.753	16.393	0.000
Intercept	1	43708.936	43708.936	8851.744	0.000	13538.230	13538.230	2835.673	0.000	26859.749	26859.749	6223.152	0.000
Gender	1	8.350	8.350	1.691	0.194	3.509	3.509	0.735	0.392	5.562	5.562	1.289	0.257
Age group	3	508.053	169.351	34.296	0.000	402.258	134.086	28.085	0.000	453.374	151.125	35.014	0.000
Gender * age group	3	17.343	5.781	1.171	0.320	6.228	2.076	0.435	0.728	6.195	2.065	0.478	0.697
Error	602	2972.610	4.938			2874.103	4.774			2598.292	4.316		
Total	610	78468.560				27605.520				49585.180			
Corrected Total	609	3527.863				3316.437				3093.565			

*R-ATC*: Right knee anterior tibial cortex, *R-PTC*: Right knee posterior tibial cortex, *R-PTAA*: Right knee proximal tibial anatomic axis

**a**. R Squared = 0.157 (Adjusted R Squared = 0.148), **b**. R Squared = 0.133 (Adjusted R Squared = 0.123), **c.** R Squared = 0.160 (Adjusted R Squared = 0.150)

**Table 4:** Post hoc analysis of the measurement values of the posterior tibial slope in the right knee with 3 different methods

		R-ATC			R-PTC			R-PTAA		
Group	N	MD	SE	*p*	MD	SE	*p*	MD	SE	*p*
Group 1 vs. Group 2	208;142	0.782^*^	0.241	0.008	0.484	0.237	0.253	0.720^*^	0.226	0.009
Group 1 vs. Group 3	208;204	1.267^*^	0.218	<0.001	1.113^*^	0.215	<0.001	1.334^*^	0.204	<0.001
Group 1 vs. Group 4	208;56	3.361^*^	0.334	<0.000	2.991^*^	0.328	<0.001	3.117^*^	0.312	<0.001
Group 2 vs. Group 3	142;204	0.485	0.242	0.277	0.630	0.238	0.051	0.613^*^	0.227	0.042
Group 2 vs. Group 4	142;56	2.578^*^	0.350	<0.001	2.507^*^	0.344	<0.001	2.397^*^	0.327	<0.001
Group 3 vs. Group 4	204;56	2.093^*^	0.335	<0.001	1.877^*^	0.329	<0.001	1.783^*^	0.313	<0.001

*Group 1*: Between 25 to 35 years old, *Group 2*: Between 36 to 45 years old, *Group 3*: Between 46 to 55 years old, *Group 4*: Between 56 to 65 years old

* The mean difference is significant at the ,05 level

**Table 5:** Measurement of posterior tibial slope using 3 different methods: correlation of measurement values of one observer at two different times and of two different observers

Variable	PTS value ICC (95% CI)			
			ICC	*p*
	Lower limit	Upper limit		
R-ATC^a^	0.968	0.990	0.982	<0.001
R-PTC^a^	0.950	0.984	0.971	<0.001
R-PTAA^a^	0.976	0.993	0.987	<0.001
R-ATC^b^	0.960	0.986	0.978	<0.001
R-PTC^b^	0.852	0.952	0.916	<0.001
R-PTAA^b^	0.976	0.992	0.986	<0.001

*R-ATC*: Right knee anterior tibial cortex, *R-PTC*: Right knee posterior tibial cortex, *R-PTAA*: Right knee proximal tibial anatomic axis

^a^ Correlation of two different measurement values of the first observer

^b^ Correlation of the measurement value of two different observers

## Discussion

The objective of this study was to investigate variations in the mean PTS angle within the Turkish population. The study aimed to assess how these variations were influenced by factors such as gender, age groups, side (right or left), and different measurement techniques. In our study, when utilizing three different measurement methods to assess PTS angles, we observed that the mean PTS angles were statistically higher in the young population. When considering the data based on age groups, our analysis revealed that the mean PTS angles were slightly higher in women and in the right knee. However, it is important to note that these differences did not reach statistical significance. Moreover, the three measurement methods used in the present study were sufficiently reliable for repeated measurements and interobserver agreement.

The PTS is defined as the angle formed by the vertical line of the tibial anatomical axis and the tangent of the tibial plateau [12]. The primary goals of successful TKA are to optimize knee joint kinematics and ensure the longevity of the implanted components [32–36]. Correct alignment of the limb and prosthesis is the most important parameter affecting the success of TKA [37–41]. The PTS angle is essential for determining the position of tibial resection in the sagittal plane TKA. Its significance lies in achieving correct alignment of the prosthesis and bones for optimal surgical outcomes [5, 6, 23–25, 38–40]. Consequently, various studies have been carried out among diverse populations, with a particular focus on evaluating variables such as gender and age. PTS angle was measured using different imaging modalities and measurement techniques. Many differences between populations have been reported in these studies [7, 42–44]. Prosthesis manufacturers recommend an mean PTS angle between 3˚ − 7˚ for TKA [10, 17]. In our study, we found the mean PTS angle of the all patients to be 8.68 ± 2.16 (SD) with the PTAA method, which was higher than the range recommended by the prosthesis manufacturers. In a Turkey study by Mısır et al. on knee MRIs, the mean PTS angle was in the range of 3˚–7˚ recommended by the manufacturers [10, 17, 29]. In a study conducted by Kaçmaz et al. in Turkey, including 1024 knee radiographs, the mean PTS angle was found to be 8.36° ± 3.3° (SD), which was higher than the recommended range by prosthesis manufacturers. This finding aligns with the results observed in our current study [27]. High PTS angles were also reported in several studies on the Chinese population [10, 24, 26]. In a limited study by Khattak et al. on 59 Pakistani patients, the mean PTS angle was 14.1° in women and 12.5° in men [15]. The findings from these studies have consistently highlighted to prosthesis manufacturers and surgeons that the PTS angle before TKA can exhibit variations based on the specific population being evaluated.

The impact of gender on the PTS angle continues to be debated. In our study, when a comparison was made between genders without considering age groups, the mean PTS angle with PTAA method was found to be lower in women than in men and the difference was statistically significant (8.55 ± 2.27 and 8.95 ± 2.2, respectively, *p* = .024). However, we suggest that the observed result may be attributed to the relatively high proportion of men within the 25–35 age range in our study. Indeed, in the comparison of genders according to age groups, we found that the mean PTS angle was higher in women than in men in all three measurement methods, but there was no statistically significant difference (*p* = .194, 0.392, and 0.257 for ATC, PTC, and PTAA, respectively) (Table 3). In their study, Kaçmaz et al. utilized X-ray imaging and employed the PTAA method to measure the PTS angles of 1024 patients. The mean PTS angle was 8.57 ± 3.4° (SD) in men and 8.16 ± 3.2° (SD) in women and the difference was statistically significant *(p* = .046) [27]. It is possible that the wide age range, spanning from 18 to 92 years, in the population of the study conducted by Kaçmaz et al. and the comparison of genders without accounting for the age factor may have contributed to the observed result. In another study, Mısır et al. evaluated knee MRI images of 1000 healthy people aged 18–50 years. In this study, medial tibial slope (MTS) and lateral tibial slope (LTS) angles were measured. Although the mean MTS (6.8°) and LTS (4.9°) levels were slightly higher in women than in men (6.5°/4.8°), no significant difference was found [29]. In a study conducted by Weinberg et al., PTS angles were measured from 1090 cadaveric tibiae using virtual representations. The findings of their study indicated that the mean PTS angles were higher in cadaveric specimens of women compared with specimens of men, and also higher in black cadaveric specimens compared with other racial groups [45]. De Boer et al. found a mean PTS angle of 8.4 ± 3.7° (SD) in their study in which they evaluated 105 cadaveric tibiae using a custom-made device. They reported that Caucasians (*n* = 34) had statistically smaller PTS angles compared with Africans (*n* = 30) (*p* < .001). However, the study did not observe any significant changes in PTS angles based on gender *(p* = .091) [42]. In a study by Khattak et al. on 59 healthy Pakistani subjects, the mean PTS angle was higher in women than in men (14.1° and 12.5°, respectively, *p =* .002) [15]. In the study conducted by Hashemi et al., which included 33 women and 22 men, the knee joint was evaluated using MRI. The researchers reported that the MTS and LTS angles were significantly larger in women compared with men (*p =* .001 and 0.002, respectively) [28]. In a study by Medda et al. including 108 patients in the East Indian population, the mean PTS angle was measured by the ATC method as 13.9 ± 3.4 (SD) in women and 13.3 ± 3.3 (SD) in men, but the difference was not statistically significant *(p =* .248) [46]. The earlier bone maturation in women and the higher prevalence of conditions like osteoarthritis and osteohyperplasia in the knee joint after the age of 40 may contribute to a larger PTS angle in women compared with men.

Despite numerous studies investigating the relationship between PTS angle and age, the reported results vary significantly [10, 27, 29, 45, 47]. In this study, the entire cohort was divided into four distinct groups based on age ranges, and a comparison of the mean PTS angles was conducted between these groups. The statistical analysis revealed significant differences in the mean PTS angles measured using the PTAA method across all age groups in this study *(p <* .05). In the ATC and PTC method, there was no significant difference between group 2 (36–45 age range) and group 3 (46–55 age range) (*p* = .242 and 0.051, respectively), while the difference between the other groups was significant *(p* < .05).

In two previous studies conducted on the Turkish population, employing different methodologies and modalities, no significant association was identified between the mean PTS angle and patient age [27, 29]. The inclusion of patients as young as 18 years old and as old as 92 years in the study by Kaçmaz et al., as well as the lack of an upper age limit in the other study, may have some limitations. Firstly, PTS angle measurements may be inaccurate due to the possibility that the development of the tibial bone is not yet complete in patients aged 18–25 years [48, 49]. The fact that the epiphyseal line is not yet closed may result in inaccurate measurement of the PTS angle. Similarly, the inclusion of patients with degenerative knee joint disease and elderly patients with osteohyperplasia of the bone in the study population may lead to higher measured mean PTS angles. A study design that takes into account both the lower and upper age limits, and potentially employs narrower age ranges, would likely offer a more accurate and focused assessment of the relationship between age and the PTS. Indeed, in the study conducted by Chen et al., where they analyzed 1257 knee radiographs from individuals aged 25–59 years, the group within the 25–29 age range exhibited a significantly higher mean PTS angle compared with the other age groups [10]. Similarly, in the study by Sun et al., involving 1431 individuals aged 0–89, the average PTS angle was significantly higher before the age of 30 compared with the 30–59 age range. The authours reported that the mean PTS angles of patients decreased with a significant difference until the age of 60 and increased again after the age of 60 [47]. In our study, although the mean PTS angles of 610 patients were measured higher in the right knee than the left knee in all three methods, no statistically significant difference was found. Some studies in which third-party evaluation was conducted support our finding [10, 15, 27, 50].

Although researchers have used other modalities in the measurement of the PTS angle, X-ray has been preferred most frequently. In addition to being a cost effective and convenient imaging modality, one of the major advantages of this modality is its ease of evaluation compared with modalities like CT and MRI. It does not require extensive expertise, and the procedure can be easily repeated as needed. The primary limitation of this examination is that it is performed in a single projection, which may restrict the comprehensive evaluation of certain anatomical structures or dimensions. In the lateral radiograph, complete overlap of the femoral condyles and overlapping of the medial and lateral tibial slopes are common. As a result, the measurement of the PTS is often performed by determining the average value of both slopes to account for this overlap. MRI and CT imaging provide more detailed information about the knee joint. However, it has the disadvantages of high cost, requiring patient compliance, temporal disadvantages and requiring considerable experience to evaluate the images. In addition, the intra and interclass correlation (ICC) values in repeated measurements are lower than in methods using X-ray [24, 28]. In our study, we measured the PTS angle with the most commonly used methods ATC, PTC, and PTAA with X-ray modality and found that the three methods differed significantly *(p* < .001). The highest mean PTS value was measured with the ATC method and the lowest mean value was measured with the PTC method and the mean difference was 4.77° in favor of ATC. In the study by Yoo et al. 90 knees of 66 women who underwent TKA were evaluated with X-ray modality and PTS angle was measured with five different measurement methods. They reported that the mean angle was 13.8° in the ATC method and 7.8° in the PTC method, with a difference of 6° [7]. In the same study, it was emphasized that the closest measurement method to the sagittal mechanical axis was the PTAA method with a difference of 0.2°. Dean et al. reported that the angle difference between the mechanical axis and PTAA was above 2° [19]. Reproducibility and high interobserver agreement are important parameters for the success of a method. In our study, although intraobserver and interobserver agreement was excellent in all three methods, the highest agreement was achieved in the PTAA method (ICC = 0.986). Previous studies have also reported high intraobserver and interobserver agreement similar to ours [1, 24, 29, 51].

Its major limitations are that degenerative arthritis patients and those over 65 were not included, and the lack of a multicenter study to better describe the Turkish population. Another limitation is that the weight and height data of the cases are missing due to the retrospective nature of the study. For this reason, the relationship between height and weight change and PTS could not be examined.

This study measured the mean PTS angle in a healthy Turkish population using three different methods. It was found that PTS differed significantly with age and the highest intraobserver and interobserver agreement among all three methods was achieved in the PTAA method. The study suggests that when selecting prosthesis for patients undergoing TKA, it is important to consider PTS angles, taking into account the patient’s age and the specific community or population they belong to.

## Data Availability

The datasets used and/or analysed during the current study available from the corresponding author on reasonable request.

## Abbreviations

ACL: Anterior Cruciate Ligament
ATC: Anterior Tibial Cortex
CT: Computed Tomography
MRI: Magnetic Resonance Imaging
MTS: Medial Tibial Slope
LTS: Lateral Tibial Slope
PACS: Picture Archiving and Communication System
PTAA: Proximal Tibial Anatomical Axis
PTC: Posterior Tibial Cortex
PTS: Posterior Tibial Slope
TKA: Total Knee Arthroplasty
